# The Infection Dynamics of Experimental *Edwardsiella ictaluri* and *Flavobacterium covae* Coinfection in Channel Catfish (*Ictalurus punctatus*)

**DOI:** 10.3390/pathogens12030462

**Published:** 2023-03-15

**Authors:** Allison L. Wise, Benjamin R. LaFrentz, Anita M. Kelly, Mark R. Liles, Matt J. Griffin, Benjamin H. Beck, Timothy J. Bruce

**Affiliations:** 1 School of Fisheries, Aquaculture, and Aquatic Sciences, College of Agriculture, Auburn University, Auburn, AL 36849, USA; 2 USDA-ARS Aquatic Animal Health Research Unit, Auburn, AL 36832, USA; 3 Department of Biological Sciences, College of Sciences and Mathematics, Auburn University, Auburn, AL 36849, USA; 4 Department of Pathobiology and Population Medicine, College of Veterinary Medicine, Thad Cochran National Warmwater Aquaculture Center, Delta Research and Extension Center, Mississippi State University, Stoneville, MS 38776, USA

**Keywords:** mixed infections, immune responses, polymicrobial, challenge

## Abstract

*Edwardsiella ictaluri* and *Flavobacterium covae* are pervasive bacterial pathogens associated with significant losses in catfish aquaculture. Bacterial coinfections have the potential to increase outbreak severity and can worsen on-farm mortality. A preliminary assessment of in vivo bacterial coinfection with *E. ictaluri* (S97-773) and *F. covae* (ALG-00-530) was conducted using juvenile channel catfish (*Ictalurus punctatus*). Catfish were divided into five treatment groups: (1) mock control; (2) *E. ictaluri* full dose (immersion; 5.4 × 10^5^ CFU mL^−1^); (3) *F. covae* full dose (immersion; 3.6 × 10^6^ CFU mL^−1^); (4) *E. ictaluri* half dose (immersion; 2.7 × 10^5^ CFU mL^−1^) followed by half dose *F. covae* (immersion; 1.8 × 10^6^ CFU mL^−1^); and (5) *F. covae* half dose followed by half dose *E. ictaluri*. In the coinfection challenges, the second inoculum was delivered 48 h after the initial exposure. At 21 days post-challenge (DPC), the single dose *E. ictaluri* infection yielded a cumulative percent mortality (CPM) of 90.0 ± 4.1%, compared with 13.3 ± 5.9% in the *F. covae* group. Mortality patterns in coinfection challenges mimicked the single dose *E. ictaluri* challenge, with CPM of 93.3 ± 5.4% for fish initially challenged with *E. ictaluri* followed by *F. covae*, and 93.3 ± 2.7% for fish exposed to *F. covae* and subsequently challenged with *E. ictaluri*. Despite similarities in the final CPM within the coinfection groups, the onset of peak mortality was delayed in fish exposed to *F. covae* first but was congruent with mortality trends in the *E. ictaluri* challenge. Catfish exposed to *E. ictaluri* in both the single and coinfected treatments displayed increased serum lysozyme activity at 4-DPC (*p* < 0.001). Three pro-inflammatory cytokines (*il8*, *tnfα*, *il1β*) were evaluated for gene expression, revealing an increase in expression at 7-DPC in all *E. ictaluri* exposed treatments (*p* < 0.05). These data enhance our understanding of the dynamics of *E. ictaluri* and *F. covae* coinfections in US farm-raised catfish.

## 1. Introduction

Aquaculture within the southeastern United States (Mississippi, Alabama, and Arkansas) is primarily dedicated to rearing channel catfish (*Ictalurus punctatus;* Rafinesque, 1818) and hybrid catfish (*Ictalurus punctatus♀* × *I. furcatus* (Valenciennes, 1840) *♂*) for food production. Aquaculture allows farmers to exert high levels of control and environmental manipulation across various production stages, which permits sustainable, high-quality, and safe catfish for consumers [1]. The southeastern economy relies heavily on catfish production, with revenues reaching $398 million in sales in 2021 [2]. With competitive profit margins for large-scale production, producers have adopted more intensive aquaculture systems, such as in-pond raceways and partitioned aquaculture systems to enhance production efficiency [3]. Though intensive production leads to increased profit yields and more efficient land use, increased stocking densities also increase the risk of disease. These risks are also exacerbated by environmental factors, such as temperature and water quality, which can increase the potential for outbreaks [4]. The catfish industry has demonstrated decades of enhancements in production methods, and fish health is a consistent target for improvement. Increased stocking densities in more intensive systems leave catfish more prone to disease, and with limited approved antibiotics or commercially available vaccines, farmers have few options for prevention and treatment [5]. 

Most economic losses in the catfish industry are attributed to bacterial disease, with disease-induced anorexia and direct losses from mortality events leading to decreased production [6]. Three bacterial pathogens, *Edwardsiella ictaluri*, *Flavobacterium covae* (formerly *F. columnare* genetic group 2) [7], and hypervirulent *Aeromonas hydrophila*, are primarily responsible for substantial economic losses throughout the sector [8,9]. *Edwardsiella ictaluri* and *F. covae* are the causative agents of enteric septicemia of catfish (ESC) and columnaris disease, respectively, which cause significant losses on farms. Diagnostic reports from the Aquatic Research and Diagnostic Laboratory (ARDL) at Stoneville, MS, over the past decade indicate a high incidence of disease associated with *E. ictaluri* or *F. covae* [10]. These pathogens have primarily been evaluated during single infections. To better manage these disease agents, the dynamics of coinfections need to be assessed. 

Generally, *E. ictaluri* has been considered a more ruinous bacterial pathogen of US farm-raised catfish [11,12]. However, columnaris disease has been a more frequent diagnosis over the past decade, accounting for 41.7% of cases submitted to ARDL compared with 32.5% for ESC from 2009 to 2019 [10]. Outbreaks of ESC typically occur when first-year fingerlings encounter the bacteria for the first time and are highly dependent upon seasonality and water temperature, with infections often in spring or fall [12,13]. Infected fish exhibit lethargy, exophthalmia, cranial ulcers, ascites, and typically display abnormal swimming behaviors, including spiral swimming patterns and stargazing [14]. Lost productivity due to morbidity and mortality culminates in an estimated $60 million in annual economic losses to the industry [15]. 

Comparably, *F. covae* is a Gram-negative bacterium responsible for columnaris disease [7]. Columnaris disease typically presents as an external infection of the skin, fins, and gills and is often in the presence of other bacterial or parasitic agents. The Louisiana Aquatic Diagnostic Laboratory in the early 1990s reported that nearly 90% of Columnaris diagnoses were mixed infections [16]. Columnaris disease, like ESC, is one of the leading causes of mortality in channel catfish [17], with losses estimated to exceed $30 million annually [8,18].

Coinfections, which occur when a host is infected with multiple pathogens, have been reported for farm-raised catfish, although information regarding prevalence, mortality rates, and mechanisms of infection is scarce [19]. Coinfections between *E. ictaluri* and *F. covae* have been documented in catfish diagnostic cases from Alabama and Mississippi [20]. While effective treatment and prevention strategies for each pathogen have been developed [21,22,23,24,25,26], the ability of these approaches to combat coinfections is unknown, and the efficacy of approved antibiotics has yet to be defined under conditions where a combination of pathogens infect a single fish. 

As a first step in defining these coinfection interactions in channel catfish, these pathogens must be assessed in tandem to determine exactly how mortality is impacted along with several innate immune parameters. At present, it is unknown if dual infections of these agents interact synergistically or antagonistically in the fish host [19], and the impact of coinfections of these two agents may be underappreciated. Herein, the dynamics of *E. ictaluri* and *F. covae* coinfections were assessed in juvenile channel catfish under controlled conditions. These studies lay the foundation for future works, assessing the pathophysiological and immunologic responses during mixed infections and the development of management strategies to minimize the impact these agents have on catfish health and production. 

## 2. Materials and Methods

### 2.1. The Bacteria and Culture Conditions

*E. ictaluri* S97-773 [27] (GenBank CP084521) was revived from cryogenic storage (−80 °C) by isolation streaking onto brain-heart infusion agar (BHIA) and incubated for 48 h at 28 °C. Following confirmation of the morphology, an individual colony was expanded in 20 mL of a brain-heart infusion broth (BHIB; BD Biosciences; Franklin Lakes, NJ, USA) for 18 h at 28 °C with shaking (175 rpm). After incubation, 100 µL of broth was used to seed 250 mL of BHIB (18 h at 28 °C, 175 rpm). The final challenge culture was adjusted to an optical density at 600 nm (OD_600_) of 1.058 using sterile BHIB and a Biophotometer Plus spectrophotometer (Eppendorf; Enfield, CT, USA). Similarly, *F. covae* ALG-00-530 [7] (GenBank MW353001) was revived from cryo-stock by isolation streaking on modified Shieh agar (MSA) [28] and underwent 24 h incubation at 28 °C. A single yellow-pigmented adherent rhizoid colony was subsequently transferred to a 50 mL conical tube containing 10 mL of sterile, modified Shieh broth (MSB), and was expanded for 12 h at 28 °C with shaking (175 rpm). An aliquot (5 mL) was used to seed 200 mL of MSB and expanded for 12 h under the same conditions. As above, the challenge culture was adjusted using sterile MSB to an OD_550_ = 0.707. Viable cell concentrations of the adjusted cultures were determined using standard plate count techniques and appropriate media for each pathogen (*E. ictaluri*: BHIA; *F. covae*: MSB). 

### 2.2. The Experimental Design (Trials A and B)

Healthy, juvenile channel catfish (Marion strain; ~15 g) from the E.W. Shell Fisheries Center at Auburn University (Auburn, AL, USA) were reared in a recirculating aquaculture system (RAS) with dechlorinated municipal water before the study initiation. To characterize coinfections involving *E. ictaluri* and *F. covae*, catfish were arbitrarily assigned to five treatment groups (6 tanks per treatment; 20 fish per tank) for in vivo infectivity trials. All fish were transferred into respective tanks containing 38 L (within a 64 L tank) two days before the challenge. The aquaria were supplied with flow-through dechlorinated municipal water at a rate of 0.5 L min^−1^ at 28 °C, with supplemental aeration. Fish were both monitored and fed twice daily during the acclimation period. Fish were randomly distributed within the tanks and treatment groups were randomly assigned to tanks. Groups 1 and 2 were exposed by immersion to the full dose of *E. ictaluri* and *F. covae*, respectively. Group 3 received a half dose of *E. ictaluri* followed by a half dose of *F. covae,* 48 h later. Conversely, Group 4 received a half dose of *F. covae* with a subsequent half dose of *E. ictaluri* after 48 h. Group 5 consisted of mock-challenged fish exposed to sterile phosphate-buffered saline (PBS; pH 7.2). The Group 5 control group received PBS twice (0 h and 48 h), just as the coinfection Groups 3 and 4, to consider any potential stress effect. Throughout the manuscript, treatments that received *F. covae* followed by *E. ictaluri* are defined as co-*F. covae*, while fish exposed to *E. ictaluri*, followed by *F. covae*, are deemed co-*E. ictaluri*. For each treatment, three tanks served to estimate challenge mortality, while three tanks were used for sampling.

During the immersion challenge, the water level was lowered to 10 L for all tanks and was restored to the normal level post-challenge. Group 1 tanks received a 6 mL inoculum (OD_600_ = 1.058) of *E. ictaluri*, bathed for 0.5 h in 10 L of rearing water (28 °C), delivering a dose of 5.4 × 10^5^ CFU mL^−1^. Group 2 tanks were dosed with a 110 mL inoculum (OD_55 0_ = 0.707) of *F. covae* for 0.5 h in 10 L, yielding a delivered dose of 3.63 × 10^6^ CFU mL^−1^. Group 3 tanks received 3 mL of the same *E. ictaluri* culture, delivering 2.7 × 10^5^ CFU mL^−1^, followed 48 h later with a 55 mL inoculum of *F. covae* culture (1.8 × 10^6^ CFU mL^−1^). Similarly, Group 4 tanks received 55 mL of *F. covae* inoculum delivering 1.8 × 10^6^ CFU mL^−1^ and subsequent 3 mL of *E. ictaluri* culture 48 h later (2.7 × 10^5^ CFU mL^−1^). All challenge doses were administered under the same conditions. Post-initiation, tanks were monitored twice daily, and the deceased fish were removed from the tanks. Mortality was used as the clinical endpoint for the trials. Feed was offered to fish twice daily, and uneaten pellets were removed at each checkpoint. Up to 20% of daily mortalities were necropsied and cultured to confirm the presence of bacteria. Coinfected groups were plated on both BHIA and MSA to culture both bacteria. The end of the challenge was determined once mortality had ceased for several days. 

A second immersion trial was conducted to include additional doses equivalent to those administered for coinfection treatments and to discern the contribution of half-doses to mortality for each pathogen. Catfish (~22 g) were distributed to 27 tanks (3 tanks per treatment; 20 fish per tank). Treatments consisted of; Full dose *E. ictaluri*, half dose *E. ictaluri*, full dose *F. covae*, half dose *F. covae*, full dose *E. ictaluri* followed by full dose *F. covae*, half dose *E. ictaluri* followed by half dose *F. covae*, full dose *F. covae* followed by full dose *E. ictaluri*, half dose *F. covae* followed by half dose *E. ictaluri,* and a sham challenge (sterile PBS). The *E. ictaluri* treatments received 4 mL (full) or 2 mL (half) of the inoculum (OD_600_ = 1.065), yielding exposure doses of 3.8 × 10^5^ CFU mL^−1^ and 1.9 × 10^5^ CFU mL^−1^, respectively. Similarly, the *F. covae* treatments received 100 (full) or 50 mL (half) inoculums of culture (OD_550_ = 0.747) which resulted in immersion baths of 7.56 × 10^6^ CFU mL^−1^ for full doses and 3.78 × 10^6^ CFU mL^−1^ for the half dose. For the challenge, catfish were bathed for 0.5 h in 10 L water, and secondary doses were delivered 48 h after the initial exposure for coinfection treatments. No sampling tanks were involved in Trial B.

### 2.3. The Collection and Sampling

Fish from Trial A were sampled (3 fish per tank and triplicate tanks per treatment group) at two, four, seven, and twenty-one days post-challenge. Fish were euthanized with a lethal overdose of buffered tricaine methanesulfonate (MS-222; Syndel, Ferndale, WA, USA) at 250 mg L^−1^. The anterior kidney, spleen, and blood were collected aseptically and used for the extraction of RNA, DNA, and serological analysis, respectively. Kidney and spleen tissues were preserved in DNA/RNA Shield^TM^ (Zymo Research Corp., Irvine, CA, USA) and stored at −20 °C until nucleic acid extraction. To assess serum lysozyme activity, fish were bled from the caudal vein using 22 ga syringes, and samples were allowed to clot overnight at 4 °C. Following separation, blood samples were concentrated at 15,000× *g* (Eppendorf 5420; Enfield, CT, USA) for 5 min, and the serum was collected by micropipette and stored at −80 °C until processing.

### 2.4. The Bacterial DNA and Tissue RNA Extraction

Reisolated bacterial colonies collected from the daily mortalities were subcultured from the posterior kidneys and spleen and were processed to extract the DNA for endpoint PCR to confirm the pathogen identity. Genomic DNA was isolated using the Omega E.Z.N.A.^TM^ Bacterial DNA Kit (Omega Bio-tek, Inc., Norcross, GA, USA), eluted with 100 µL of provided elution buffer, quantified spectrophotometrically (Nanodrop One^c^; Thermo Fisher Scientific, Waltham, MA, USA), and stored at −20 °C until PCR analysis. Kidney tissue samples, harvested at all time points, were manually homogenized in the DNA/RNA Shield^TM^ (Zymo Research Corp., Irvine, CA, USA) using a mortar and pestle. RNA was extracted following the Zymo Research Quick-RNA^TM^ MiniPrep Plus kit, eluted with 100 µL of nuclease-free water, quantified spectrophotometrically, and stored at −80 °C. 

### 2.5. The Gene Expression Analysis

The extracted RNA was diluted to 50 ng µL^−1^ using nuclease-free water and converted to cDNA using the High-Capacity cDNA Reverse Transcription Kit™ (Applied Biosystems, Waltham, MA, USA), following the manufacturer’s instructions. Each 20-µL reaction contained 2 µL of 10× RT buffer, 0.8 µL of 25× dNTP Mix, 2 µL of 10× RT random primers, 1 µL of MultiScribe™ reverse transcriptase, 500 ng of template RNA, and nuclease-free water to volume. cDNA was synthesized in a MiniAmp Plus thermal cycler (Applied Biosystems, Carlsbad, CA, USA) programmed for one cycle of 25 °C for 10 min, 37 °C for 120 min, and 85 °C for 5 min, and was subsequently diluted to 2.5 ng µL^−1^ using nuclease-free water. Four genes were evaluated for expression analysis, namely *il1β*, *tnfα* [29], *il8* [30], and *tgfβ-1* [31]. The housekeeping genes, *ef1a* [30] and *actb* [32], were used for normalization. The PCR was carried out in 10-µL volumes consisting of 5 µL PowerUp SYBR Green Master Mix™ (Applied Biosystems, Carlsbad, CA, USA), forward and reverse primers at 500 nM (Supplemental Table S1), and 2 µL of sample cDNA nuclease-free water to volume. Each sample was run in duplicate along with no-template controls consisting of nuclease-free water in place of the template cDNA. PCRs were run on a QuantStudio™ 5 Real-Time PCR system (Applied Biosystems Carlsbad, CA, USA) programmed for initial steps of 50 °C for 2 min and 95 °C for 2 min, followed by 40 cycles of 95 °C for 15 s, 58 °C for 15 s, and 72 °C for 30 s, with data collection occurring after the 72 °C elongation. For each gene target, reaction efficiencies were assessed using serial dilutions of cDNA covering five orders of magnitude, run in duplicate, and starting at 10 ng. For each gene, reaction efficiencies ranging from 90–110% were considered acceptable [33]. For calculations, the 2^−ΔΔCt^ method was implemented [34], taking into consideration the combination of both housekeeping genes along with the control group for each time point. Thus, each fold change of the gene of interest was expressed relative to that of the control group average at that time point.

### 2.6. The Lysozyme Activity Assay

Lysozyme activity was ascertained by comparisons to prepared standards, following previously published protocols [35]. The standards consisted of dilutions of a stock 480 µg mL^−1^ chicken lysozyme egg white (Rockland Immunochemicals, Pottstown, PA, USA) dissolved in sodium phosphate buffer (SPB; 0.04 M Na_2_HPO_4_; pH 6.0) and diluted to create a standard curve with a range of 0–16 µg mL^−1^. Freeze-dried *Micrococcus lysodeikticus* (Worthington Biochemical, Lakewood, NJ, USA) was resuspended at 0.25 mg mL^−1^ with SPB, and 250 µL of the bacterial suspension was added to each well, along with 10 μL of sera. Each sample was run in duplicate. Absorbances at OD_450_ were collected after a 20 min incubation at 37 °C with Synergy HTX Multimode Reader (BioTek, Winooski, VT, USA) and compared with concurrently run standards.

### 2.7. The PCR Confirmation of Recovered Isolates

The identity of presumptive *E. ictaluri* isolates recovered from the dead or moribund fish was confirmed by *E. ictaluri*-specific PCR. All the PCR testing was conducted on a MiniAmp thermal cycler (Applied Biosystems, Carlsbad, CA, USA). Colony PCR was performed on representative colonies to confirm the presumptive identification as *E. ictaluri.* Specific ESC primers (ESCF and ESCR) [36] were used. Each 25-µL reaction consisted of a 12.5 µL 2× hot-start PCR-to-gel-master mix (Amresco LLC, Solon, OH, USA), 0.2 mM of each primer, and nuclease-free water to volume. Positive (DNA extracted from *E. ictaluri* (S97-773)) and negative controls (nuclease-free water) were run in tandem with the samples. Cycle conditions were 95 °C for 5 min, followed by 30 cycles at 95 °C for 15 s, an annealing temperature of 58 °C for 15 s, and 72 °C for 15 s. The final extension was run at 72 °C for 5 min. Aliquots of the PCR products (5 µL) were separated by electrophoresis through 2.0% agarose gels in a Tris-acetate-EDTA (TAE) buffer, stained with GelRed (Biotium Inc., Fremont, CA, USA), and were visualized by ultraviolet transillumination in a Gel Doc Go imaging system (Bio-Rad, Inc., Hercules, CA, USA). Samples were run alongside concurrently run molecular weight standards to confirm the presence of appropriately sized bands.

Presumptive *F. covae* recovered from dead fish were confirmed by multiplex PCR as described by [7,37]. Each 25 μL reaction contained a 12.5 µL 2× hot-start PCR-to-gel-master mix (Amresco LLC, Solon, OH, USA), 2 µL of the primer cocktail (0.5 µM GG-forward, 0.1 µM GG1-reverse, 0.45 µM GG2-reverse, 0.45 µM GG3-reverse, 0.3 µM GG4-reverse), 9.5 µL of nuclease-free water, and 1.0 µL of template DNA. The cycle parameters used were: 95 °C for 5 min, 40 cycles of 94 °C for 30 s, 56 °C for 20 s, and 72 °C for 1 min, followed by 10 min at 72 °C. The *F. covae* AL-02-36^T^ type strain was run as a positive control. As described above, PCR products (5 µL) were resolved on a 2.0% agarose gel via electrophoresis. 

### 2.8. Statistical Analyses

Comparisons between treatment groups over time for cumulative percent mortality, lysozyme activity (2, 4, and 7 DPC), and gene expression analyses (2, 4, and 7 DPC) were performed using a two-way repeated measures ANOVA (α = 0.05) for treatment, time, and treatment × time, with tanks included as a random factor. Serum lysozyme activity was analyzed separately at 21 DPC using a one-way ANOVA due to a lack of surviving fish within sampling tanks, so in some instances, mortality observation tanks were used in place. Tukey’s post hoc test was conducted when treatment effects were significant (*p <* 0.05). Statistical analysis was performed using R statistical software (R core Team, 2021). All errors reported throughout the paper represent the standard error of the mean among treatment tanks, as tanks were defined as the experimental unit.

## 3. Results

### 3.1. Infectivity Trial A

**Figure 1:**
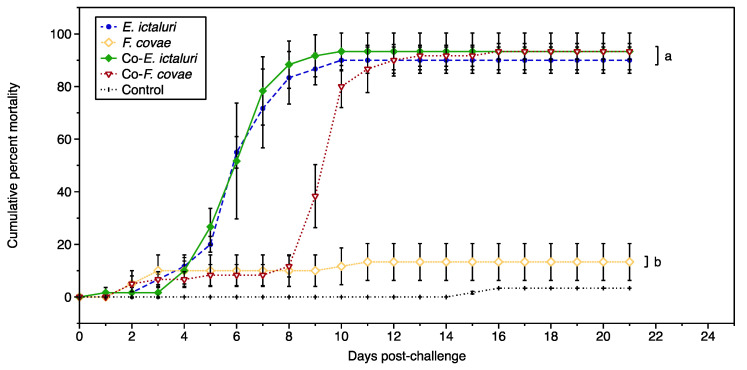
The cumulative percent mortality due to single infections of *E. ictaluri* and *F. covae* and co-infections from both pathogens over the entirety of the trial (21 days). Letters represent significance within treatment groups. Each treatment group had three tanks (*n* = 3). The bars represent the standard error of the mean for each day.

Daily mortality was recorded across the triplicate tanks over 21 days (Figure 1). The cumulative percent mortality (CPM) of the *E. ictaluri*-only group (90.0 ± 4.1%) or the two coinfection treatments (co-*E. ictaluri;* 93.3 ± 5.4%; co-*F. covae;* 93.3 ± 2.7%) was significantly different from the CPM of the *F. covae* only group (13.3 ± 5.9%; *p* < 0.001), indicating that the mortality observed in this trial was primarily due to the *E. ictaluri* infection. The onset of mortality in the co-*F. covae* was delayed compared with treatments receiving *E. ictaluri* alone or first. Fish exposed to *E. ictaluri* alone or followed with *F. covae* infection first showed signs of illness such as lethargy, reduced feeding response, and exophthalmia and mortality four to six days post-challenge, while fish exposed to *F. covae* followed by *E. ictaluri* challenge 48 h after *F. covae* exposure did not show signs of disease until 9 days post-challenge, although the CPM of any treatment exposed to *E. ictaluri* was not significantly different (*p* > 0.05) (Figure 1).

**Figure 2:**
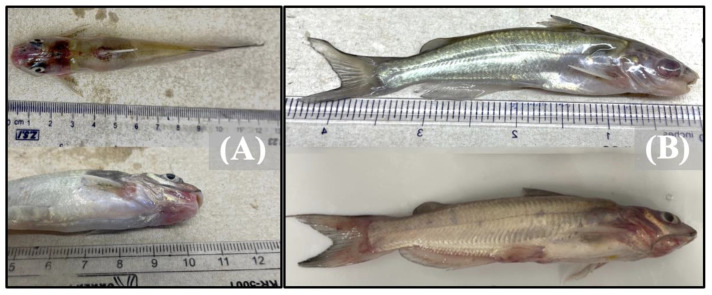
Images depicting catfish with clinical signs due to (**A**) co-infection with *E. ictaluri* first, then *F. covae* 48 h post-initial inoculation, exhibiting both saddleback lesions, discoloration, and external hemorrhaging (**B**) infection only with *E. ictaluri*, exhibiting ocular and fin hemorrhaging and exophthalmia.

The fish exposed solely to *E. ictaluri* exhibited exophthalmia, petechial hemorrhaging of the pectoral and anal fins, internal hemorrhaging, and eye hemorrhaging (Figure 2).

**Figure 3:**
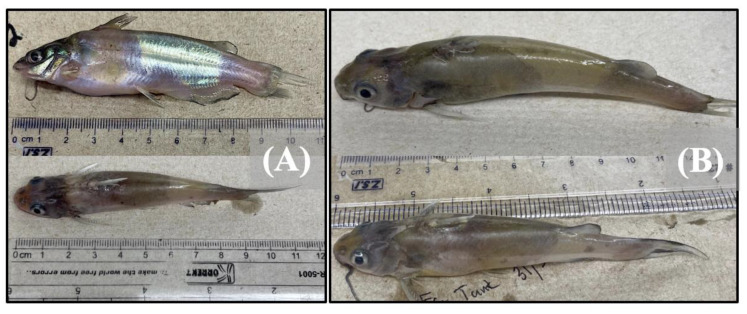
Images depicting catfish with clinical signs due to (**A**) co-infection with *F. covae* first, then *E. ictaluri* 48 h post-initial inoculation, or (**B**) infection only with *F. covae*.

Fish exposed to *F. covae* presented saddleback lesions along the dorsal fin, characteristic of columnaris disease, and exhibited internal hemorrhaging of the intestines and anterior kidneys (Figure 3). Coinfected fish from both treatment combinations demonstrated a mix of single infection clinical signs, exhibiting saddleback lesions and intestinal or ocular hemorrhaging (Figure 2A and Figure 3B). Fish exposed to both bacterial pathogens contained both *E. ictaluri* and *F. covae* bacterial colonies, while bacterial colonies recovered from treatment groups exposed to only single pathogens presented only colonies from the pathogen to which they were exposed.

### 3.2. Infectivity Trial B

**Figure 4:**
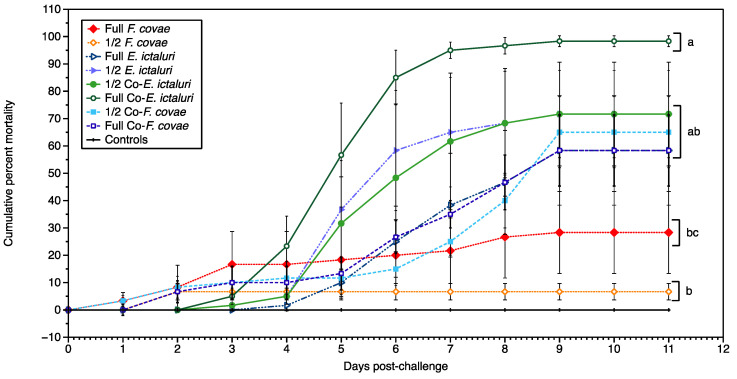
The cumulative percent mortality from Trial B due to single infections of *E. ictaluri* or *F. covae* and co-infections from both pathogens over the entirety of the trial (11 days). Letters represent significance within treatment groups. Each treatment group had three tanks (*n* = 3). Bars represent the standard error of the mean for each day.

Trial B included half-doses across the treatment groups and was conducted identically to Trial A (Figure 4). Treatment groups showed significant differences in the CPM (*p* < 0.001). Fish exposed to *F. covae* alone averaged a CPM of 28.3 ± 11.9%. Mortality in fish administered a half-dose of *F. covae* (6.7 ± 2.7%) was not significantly different (*p* > 0.05) compared with the full *F. covae* dose, due to a high level of variability between replicates. Mortality comparisons between full- and half-doses for each treatment group were also insignificant. The CPM (98.3 ± 1.4%) for the full dose *E. ictaluri/F. covae* treatment group was significantly different from the CPM of the full *F. covae* dose (28.3 ± 11.9%; *p <* 0.01), and the half dose of *F. covae* (6.7 ± 2.7%; *p* < 0.001). Clinical signs during Trial B were consistent with Trial A; however, fish exposed to *F. covae* and then *E. ictaluri* presented solely with saddleback lesions with mild external hemorrhaging along with distended abdomens.

### 3.3. Serum Lysozyme Activity

**Figure 5:**
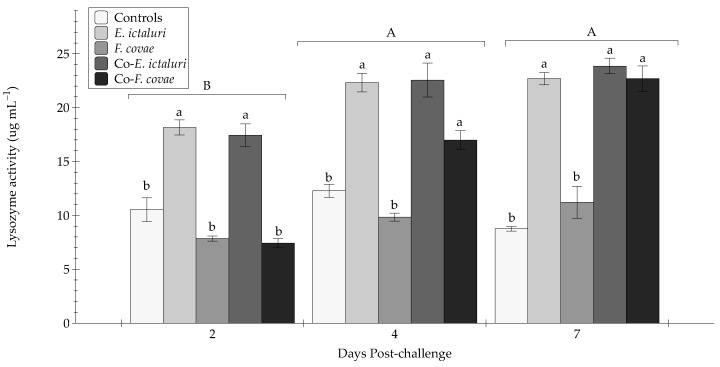
The lysozyme activity (µg mL^–1^) in sera from sampled fish at two, four, and seven days post-challenge. Each treatment group was conducted in triplicate (*n* = 3). Capital letters indicate significant differences in activity between treatment periods, and lowercase letters represent significance within treatment groups. Error bars represent the standard error of the mean for each treatment group.

Serum lysozyme activity from Trial A was evaluated at two, four, and seven days post-challenge (Figure 5). Comparisons were made amongst the treatment groups at and between the time points. Interactions between time and treatment were significant (*p* < 0.001).

**Figure 6:**
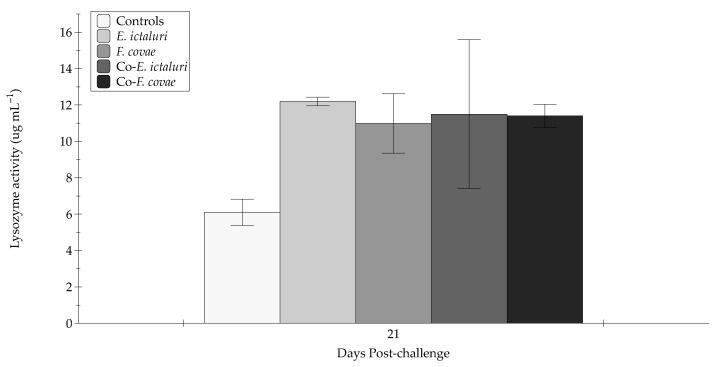
The lysozyme activity (µg mL^–1^) in sera from sampled fish at 21 days post-challenge. Each treatment group was conducted in triplicate (*n* = 3). Error bars represent the standard error of the mean for each treatment group.

At 2 DPC, lysozyme activity was significantly elevated for groups exposed to *E. ictaluri* (co-*E. ictaluri* and *E. ictaluri*) compared with controls (*p* < 0.001). Further, the *E. ictaluri* and co-*E. ictaluri* treatment groups demonstrated significantly (*p* < 0.001) greater lysozyme activity than the co-*F. covae* and *F. covae* treatment groups. Similar results were observed at 4 DPC, although with an increase in lysozyme activity compared with 2 DPC (*p* < 0.001). Again, co-*E. ictaluri* and *E. ictaluri* treatment groups exhibited significantly greater (*p* < 0.001) lysozyme activity compared with the *F. covae* treatment and controls (*p* < 0.001). Lysozyme activity significantly increased between 2 and 4 DPC for the co-*F. covae* treatment but was not statistically different from the co-*E. ictaluri* or *E. ictaluri* treatment groups (*p* > 0.05). For all groups, lysozyme activity at 7 DPC was similar to 4 DPC, yet greater than 2 DPC (*p* > 0.05). Again, activity in the *E. ictaluri*, co-*E. ictaluri*, and co-*F. covae* treatment groups were greater than the *F. covae* and control (*p* < 0.001) groups. Peak lysozyme activity occurred at 4 and 7 DPC. When analyzed with all other times, a significant decrease in lysozyme activity at 21 DPC was observed when compared with both 4 and 7 DPC (*p* < 0.001). Due to a lack of surviving fish within samplings tanks, 21 DPC lysozyme activity was analyzed separately, and no significance was determined between treatment groups (*p* > 0.05) (Figure 6).

### 3.4. Gene Expression Analysis

**Figure 7:**
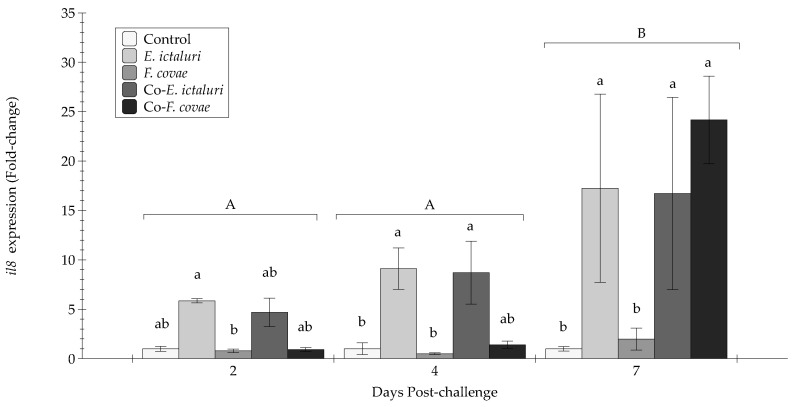
The *il8* expression (fold-change) was evaluated from extracted anterior kidneys two, four, and seven days post-challenge. Each treatment group was conducted in triplicate (*n* = 3). Capital letters indicate significant differences in quantity between treatment periods, and lowercase letters represent significance within treatment groups. Error bars represent the standard error of the mean for each treatment.

At 2 DPC, single *E. ictaluri* had elevated *il8* expression compared with single *F. covae* (*p* < 0.05) (Figure 7). At 4 DPC, *E. ictaluri* and co-*E. ictaluri* treatment groups exhibited a greater *il8* gene expression than all other groups (*p* < 0.05). At 7 DPC, *il8* expression peaked, with *E. ictaluri*, co-*E. ictaluri*, and co-*F. covae* treatment groups yielded greater *il8* expression than the *F. covae* treatment and controls (*p* < 0.01). 

**Figure 8:**
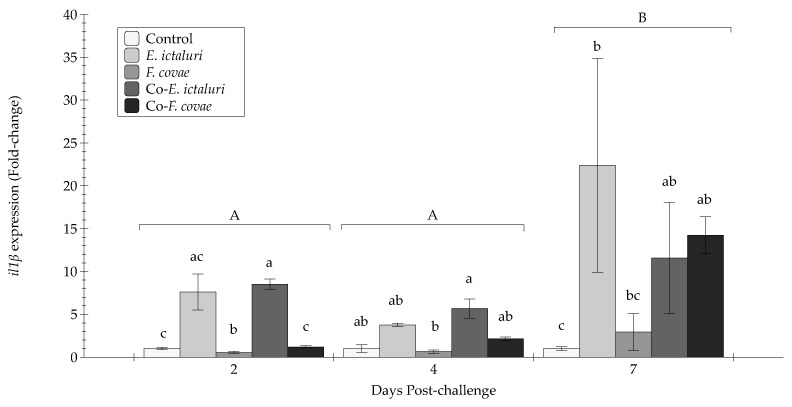
The *il1β* expression (fold-change) was evaluated from extracted anterior kidneys during two, four, and seven days post-challenge. Each treatment group was conducted in triplicate (*n* = 3). Capital letters indicate significant differences in quantity between treatment periods, and lowercase letters represent significance within treatment groups. Bars represent the standard error of the mean for each treatment.

At 2 DPC, the *il1β* expression for *E. ictaluri* and co-*E. ictaluri* treatment groups were increased compared with *F. covae* and co-*F. covae* groups as well as controls (*p* < 0.01) (Figure 8). Similar levels of expression were observed at 4 DPC, although no significant differences existed between the treatments or controls. Similar to *il8*, there was increased *il1β* expression at 7 DPC compared with 2 and 4 DPC (*p* < 0.001), with the *E. ictaluri* treatment exhibiting greater *il1β* expression than the *F. covae* treatment and control groups (*p* < 0.05). The co-*F. covae*, and co-*E. ictaluri* treatment groups also exhibited significantly greater *il1β* expression than the control group (*p* < 0.01). 

**Figure 9:**
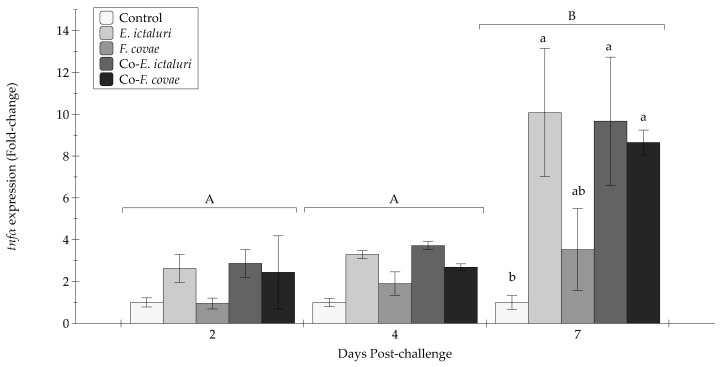
The *tnfα* expression (fold-change) was evaluated at two, four, and seven days post-challenge. Each treatment group was conducted in triplicate (*n* = 3). Capital letters indicate significant differences in quantity between treatment periods, and lowercase letters represent significance within treatment groups. Bars represent the standard error of the mean for each treatment.

**Figure 10:**
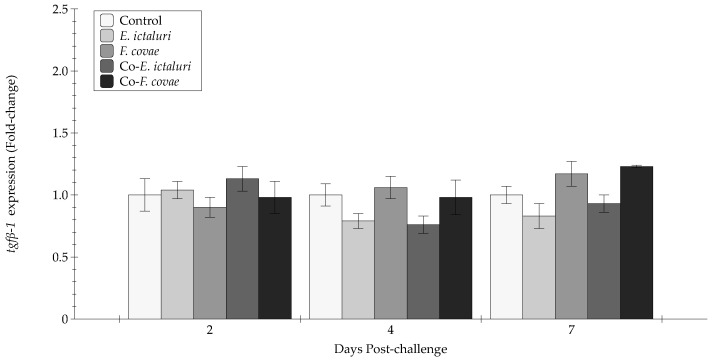
The *tgfβ-1* expression (fold-change) was evaluated from extracted anterior kidneys during two, four, and seven days post-challenge. Each treatment group was assessed in triplicate (*n* = 3). Bars represent the standard error of the mean for each treatment.

There were no statistical differences in the expression of *tnfα* at 2 and 4 DPC (Figure 9). At 7 DPC, *tnfα* was increased in the co- *E. ictaluri*, co- *F. covae*, and *E. ictaluri* treatment groups compared with unexposed controls (*p* < 0.001). There were no significant differences in the expression of *tgfβ-1* throughout the experiment (Figure 10). Interactions between time and treatment for each gene (*il1β*, *tgfβ-1*, *tnfα*, *il8*) were evaluated and were not significant (*p* > 0.05).

## 4. Discussion

Given the prevalence of *E. ictaluri* and *F. covae* throughout US catfish aquaculture, the synergistic dynamics of these two pathogens must be evaluated to appreciate the impact of coinfections on fish health [38]. Concurrent infections are prevalent throughout aquaculture industries and occur with a variety of different pathogens [39]. Tilapia (*Oreochromis niloticus*; Linnaeus, 1758), zebrafish (*Danio rerio*; F. Hamilton, 1822), rainbow trout (*Oncorhynchus mykiss*; Walbaum, 1792), Atlantic salmon (*Salmo salar*; Linnaeus, 1758), koi (*Cyprinus rubrofuscus*; Lacépède, 1803), shrimp (suborder Caridea; Dana, 1852), and oysters (family Ostreidae; Rafinesque, 1815) all experience coinfections that can augment mortality [40,41,42,43]. Within catfish species, coinfections of *A. hydrophila* and *E. ictaluri* can increase mortality [44]. Though coinfections commonly occur, information on pathogenicity and host response is virtually unknown, as most research has focused on single pathogen infections [45,46]. Additionally, this information is not frequently investigated or reported within the U.S. catfish industry. In both infectivity trials and previous coinfection work, *E. ictaluri* acts as the primary driver for mortality. In contrast, *F. covae*, though it causes mortality, acted more as a secondary pathogen within this challenge model. The co-*E. ictaluri* group did not demonstrate a difference in mortality compared with *E. ictaluri* alone, while the co-*F. covae* group displayed significantly higher mortality than the single dose of *F. covae* in trial A, presumably due to the introduction of *E. ictaluri.* In trial B, the co-*E. ictaluri* group again exhibited higher mortality than observed for all single-infected treatment groups. Previous trials, evaluating coinfections associated with *E. ictaluri,* reported high mortality levels in *E. ictaluri*-only groups [47]. Crumlish et al. (2010) observed high mortality (80%) caused by *E. ictaluri*, whereas *A. hydrophila* induced very low mortality (10%). Comparatively, coinfection with the two yielded 100% mortality, with much of that likely driven by *E. ictaluri* [44]. A culmination of previous coinfection trials using *E. ictaluri* and the data presented herein offers strong evidence that *E. ictaluri* is the primary pathogen under these experimental conditions. Still, it would be of interest to repeat this trial work with additional *F*. *covae* strains and additional pathogen implementation time points to better discern the contributions of each pathogen to the observed mortality. 

In addition to assessments of cumulative mortality, there were differences in the onset of mortality, depending on which pathogen the fish were exposed to first. In the first trial, differences in the day of the first fatality appeared driven by *E. ictaluri*, with delayed fatality in fish challenged later with *E. ictaluri*. However, in the second trial, the treatment group challenged with a full dose of co-*E. ictaluri* demonstrated 98.33% CPM, while the comparable full dose of the co-*F. covae* group averaged 58.33% CPM. Although not different, this was an interesting decrease observed due to varying the timing of pathogen inoculation. This difference in onset and severity of disease between the alternation of *E. ictaluri* and *F. covae* has not previously been documented. These results suggest the possibility of potential antagonistic interactions between the pathogens depending on which pathogen the fish were exposed to first. Such antagonistic interactions may cause pathogens within the host to compete for resources, thus lessening the effects of one pathogen and causing a decrease in mortality [48]. Obtaining a complete understanding of coinfection interactions may better define the primary and secondary roles of each pathogen concerning virulence, and help in developing more effective treatments and mitigation strategies, especially for species raised in natural, open pond environments [20,41]. Further histopathological assessments are needed to confirm and better characterize the clinical differences in co- versus single infections, as they are crucial in assessing disease [49]. 

Lysozymes within diseased channel catfish serve as one of the first immunological host defenses [50]. Within fish, lysozymes are present in the mucosal barrier and sera of fish [51]. The increases in lysozyme activity observed in the coinfected channel catfish, relative to single-infected fish, provide further evidence that coinfections can drastically upregulate the host’s innate immune response, thus giving insight into which innate immune parameters are enhanced due to infection [52]. Lysozyme activity within all groups followed the same pattern as mortality between treatment groups. While monitoring lysozyme activity between groups over 21 days it was observed that each treatment follows the same trend, with lysozyme activity low at 2 DPC, increasing to maximum observed activity levels at 4 and 7 DPC, then declining at 21 DPC. The increase in lysozyme activity documented in *E. ictaluri*, co- *E. ictaluri*, and co- *F. covae* corresponds with the mortality observed within each group, suggesting that *E. ictaluri* specifically contributed to the observed mortality and increased lysozyme activity. When evaluating enzyme activity between periods (2, 4, 7, 21 DPC), 2 DPC and 21 DPC had no significant difference, coinciding with the infection’s beginning and end. At 21 DPC, lysozyme activity dramatically decreased, indicating downregulation of the innate immune components and further suggesting that surviving fish have cleared the infection. Other studies have documented lysozyme activity with *E. ictaluri* and *F. covae* during single infections. Ren et al. (2015) observed catfish exposed to *E. ictaluri* caused a substantial increase in lysozyme expression within major internal organs (liver, spleen, and kidney) and the mucosal surface of the fish [53]. Similar results were demonstrated within these disease trials in this study. Interestingly, lysozyme activity seems to follow the expression patterns of an *E. ictaluri* infection during coinfections. In trial B, co-*F.covae* treatment had lower mortality than the co-*E. ictaluri* treatment group, but lysozyme levels appeared to increase in response to *E. ictaluri*. Monitoring lysozyme presence throughout illness provides an understanding of host immunological processes in response to specific pathogens like *E. ictaluri*. Due to lysozymes possessing antibacterial properties in the mucosa, liver, and intestinal tract [54], lysozyme activity may, to some degree, mitigate disease [55]. But in this study, lysozyme activity was correlated with fish with a severe *E. ictaluri* infection and high ultimate mortality. Thus, further experimentation would help to understand the role of lysozymes (both serum and mucus) in targeting specific catfish pathogens via the host immune response.

All pro-inflammatory cytokine genes (*il8*, *tnfα*, and *il1β)* followed the same trend, being prominently increased leading up to 7 DPC. There were concerning expression changes over time; at 2 and 4 DPC, each immune gene demonstrated no significant differences in expression, while at 7 DPC, each gene had a significant upregulation, indicating that channel catfish have an upregulation of innate pro-inflammatory genes to combat bacterial pathogens during infection. Treatment groups *E. ictaluri*, co-*E. ictaluri*, and co-*F. covae* had significantly more gene expression at day 7 than both *F. covae* and the controls. This also indicates that the higher the mortality experienced by the treatment, the higher the level of expression for each of the pro-inflammatory genes. However, *tgfβ-1* expression had no significant differences between the treatment groups or sampling periods, likely due to the function of *tgfβ-1* as an immunosuppressive cytokine that inhibits the immune response. During infection, the immune response will upregulate genes that will aid in the fish’s survival, so the upregulation of *tgfβ-1* would be counterproductive. To better discern the impacts of bacterial coinfection, further studies evaluating the link between the pathogen and host response (i.e., transcriptomics) may allow researchers to discern the individual contribution of each pathogen on the host cytokine response. Additionally, the simultaneous influence of two bacteria may also produce exhaustion of the host metabolism, which may have a role in cytokine expression dynamics. 

In summary, a major conclusion of this study was that a combined *E. ictaluri* and *F. covae* infection increased fish mortality. Within these experimental conditions, *E. ictaluri* acted as the primary driver of mortality in both trials. While *F. covae* alone resulted in low mortality, when combined with *E. ictaluri*, this pathogen caused a substantial increase in mortality. During co- or single infection with *E. ictaluri*, an upregulation of lysozyme activity and pro-inflammatory cytokines was observed. Though several characteristics were evaluated during *E. ictaluri* and *F. covae* coinfections, future studies are needed to resolve the respective roles of each bacterial pathogen and how specific virulence factors impact host immune and other responses. A more natural coinfection disease model can aid fish health diagnosticians and channel catfish producers to better control bacterial coinfections with more rapid and accurate disease diagnostics and develop more efficient treatments that consider the presence of multiple pathogens. 

## Data Availability

The data that support the findings of this study are available from the corresponding author upon reasonable request.

## Supplementary Materials

The following supporting information can be downloaded at: https://www.mdpi.com/article/10.3390/pathogens12030462/s1, Table S1: Listing of primer sets used in the study for PCR and qPCR analyses.
